# A Polarization-Insensitive and Wide-Angle Terahertz Absorber with Ring-Porous Patterned Graphene Metasurface

**DOI:** 10.3390/nano10071410

**Published:** 2020-07-19

**Authors:** Hongyang Shen, Fengxiang Liu, Chunyang Liu, Dong Zeng, Banghong Guo, Zhongchao Wei, Faqiang Wang, Chunhua Tan, Xuguang Huang, Hongyun Meng

**Affiliations:** Guangdong Provincial Key Laboratory of Nanophotonic Functional Materials and Devices, School of Information and Optoelectronic Science and Engineering, South China Normal University, Guangzhou 510006, China; hongyangshen@m.scnu.edu.cn (H.S.); fengxiangliu@m.scnu.edu.cn (F.L.); chunyangliu@m.scnu.edu.cn (C.L.); dongzeng@m.scnu.edu.cn (D.Z.); guobangh@163.com (B.G.); wzc@scnu.edu.cn (Z.W.); fqwang@scnu.edu.cn (F.W.); tch@scnu.edu.cn (C.T.); huangxg@scnu.edu.cn (X.H.)

**Keywords:** terahertz, graphene, broadband absorber, metasurface

## Abstract

A broadband terahertz (THz) absorber, based on a graphene metasurface, which consists of a layer of ring-porous patterned structure array and a metallic mirror separated by an ultrathin SiO_2_ dielectric layer, is proposed and studied by numerical simulation. The simulated results show that the absorptivity of the absorber reaches 90% in the range of 0.91–1.86 THz, and the normalized bandwidth of the absorptivity is 68.6% under normal incidence. In the simulation, the effects of the geometric parameters of the structure on the absorption band have been investigated. The results show that the absorber is insensitive to the incident polarization angle for both transverse electric (TE) and transverse magnetic (TM) under normal incidence. In addition, the absorber is not sensitive to oblique incidence of the light source under TE polarization conditions, and has an approximately stable absorption bandwidth at the incident angle from 0° to 50°. The absorption band can be adjusted by changing the bias voltage of the graphene Fermi level without varying the nanostructure. Furthermore, we propose that a two-layer graphene structure with the same geometric parameters is separated by a dielectric layer of appropriate thickness. The simulated results show that the absorptivity of the two-layer absorber reaches 90% in the range of 0.83-2.04 THz and the normalized bandwidth of the absorptivity is 84.3% under normal incidence. Because of its excellent characteristics based on graphene metamaterial absorbers, it has an important application value in the field of subwavelength photonic devices.

## 1. Introduction

The area between the far infrared and the ultra-microwave is terahertz (THz), whose frequency ranges from 0.1 to 10 THz. THz waves have important application significance. It can be used in imaging, drug inspection, biological detection, and intracellular protein composition analysis [1,2,3,4]. In recent years, terahertz absorbers have generated much research interest due to their unique properties and potential applications [5]. Since being proposed by Landy et al. [6], metamaterial absorbers have greatly attracted the interest of researchers, and many types have been reported. The most typical metamaterial absorber consists of a metal/dielectric/metal three-layer sandwiched microstructure [7,8,9,10]. To obtain a wide absorption, multi-layer graphene/metal coupled metamaterial absorbers have been proposed [11,12,13,14]. However, these structured absorbers cannot be adjusted after manufacturing, and their structures are complex and difficult to manufacture [15]. Because the surface conductivity can be controlled by the external load voltage, graphene has obvious advantages over traditional precious metals, such as gold and silver, which has made graphene-based absorbers a research hotspot in the field of plasma photonic device design. Compared to a traditional metal absorber, the graphene metamaterial absorber has simple structure and tunability [16,17]. The design of dual-band or multi-band absorbers obtained from the basic resonance overlap of conventional single-band absorbers has also received much attention [18,19,20]. The complicated structure and narrow absorption bandwidth limits their applications [21,22,23]. The absorption bandwith can be enlarged with the mutual coupling of multilayer graphene/metal [24,25,26], and several graphene-based absorbers with simple structures have also been proposed [27,28,29,30]. Although the fractional bandwidth of absorbers with metal structures or multi-layer graphene structures can be further improved, most of them have disadvantages such as difficulty in tuning the absorption band and sensitivity to polarization angles and incident angles. Therefore, it is very beneficial to study the novel graphene-type terahertz absorber with a wide absorption band, as well as polarization insensitivity, multi-angle incidence, and tunability.

In this paper, we propose a polarization-independent and wide-angle absorber based on a graphene metasurface at terahertz frequencies, which consists of a graphene array structure with ring-porous patterns, a dielectric layer and a metal layer to achieve a high-bandwidth absorption. The metal layer is used as the bottom-most layer and the dielectric layer is used as an intermediate layer. The patterned graphene array is periodically arranged on the dielectric layer silicon dioxide. The simulated results show that more than 90% of the broadband terahertz absorption in the range of 0.91 to 1.86 THz can be achieved. By optimizing the parameters of the absorber, a fractional bandwidth of 68.6% can be achieved under normal incidence and graphene Fermi level of 0.7 eV. Due to the axial symmetry structure, the absorber has insensitive polarization characteristics for the transverse electric (TE) and transverse magnetic (TM) polarized terahertz waves under normal incidence. For the TE polarization, the absorber has a relatively stable absorption bandwidth in the incident angle range from 0° to 50°. Finally, we study the tunability of the absorption band of the structure. By changing the graphene Fermi energy from 0.7 eV to 0.2 eV, the absorption rate of the absorber can be adjusted. Furthermore, a double sandwich graphene structure has also been studied, and the simulated result indicates that a wider absorption bandwidth (from 0.91 THz to 1.21 THz) and big fractional bandwidth (from 68.6% to 84.3%) can be achieved. This study provides new inspiration for the design of graphene-based tunable wideband absorbers that can be used in terahertz optoelectronic devices such as photodetectors and biosensors.

## 2. Design of Structure

**Figure 1:**
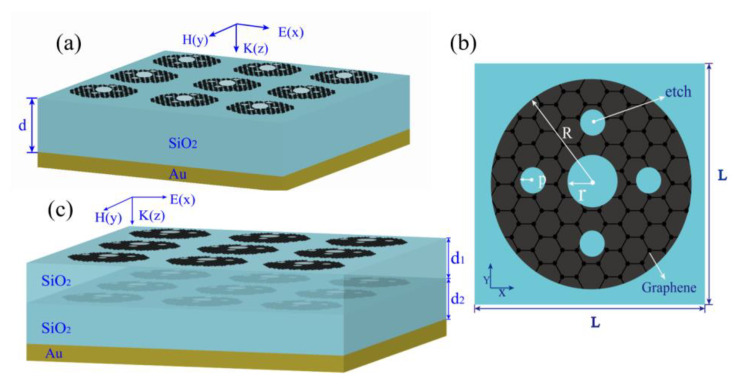
(**a**) Schematic diagram of an absorber composed of toroidal porous graphene, medium, and Au. (**b**) Unit cell of the absorber, in which the values of *R*, *r*, and *p* are 6.5 μm, 2.05 μm, and 1.3 μm, respectively. (**c**) Double-layer graphene metasurface structure, in which the value of *d*_1_ and *d*_2_ are 12 μm and 16 μm, respectively.

The structure of the absorber is shown in Figure 1a, and the planar structure of one unit is shown in Figure 1b. The absorber comprises a three-layer structure with a single-layer patterned graphene-dielectric silica-metal reflective plate. The top layer is a graphene sheet with a ring-porous pattern arranged periodically, where *R*, *r*, and *p* denote the length of the outer and inner radius of the graphene ring and the radius of the small circle around the ring, respectively. The center of the small circle is at the center of the graphene ring. *L* refers to the width and length of a periodic unit along the x and y direction, which are both 15 μm. The middle dielectric layer comprises 28 μm-thick non-destructive SiO_2_ with a dielectric constant *ε* = 3.9 [31]. In the simulations, a layer of gold with a thickness of 0.5 μm and a conductivity of 4.09 × 10^7^ S/m was used as the bottom metal layer, which is thick enough to meet the typical skin depth in the THz range. Therefore, wave transmission was completely suppressed [32].

Graphene is a two-dimensional honeycomb planar material with carbon atoms. Graphene supports surface plasmon resonance (SPR) in the infrared and terahertz bands [33]. Because of its high carrier mobility and graphene doped in a broadband region or with a regular structure pattern, it can effectively enhance light absorption [34]. One of the most important characteristics of graphene is that its Fermi level can be freely adjusted by applying an electrostatic bias without changing the geometrical structure to reconstruct new structures [35]. In the simulation, the graphene layer we describe is characterized by an effective surface conduction model. It is known that the surface conductivity of graphene is described by the Kubo formula [36], which considers both the intra-band transitions and the inter-band transitions as follows:

(1) $$\sigma_{gra}=\sigma_{intra}+\sigma_{inter}=\frac{2e^{2}k_{B}T}{\pi \hbar^{2}}\frac{i}{\omega +i/\tau }\ln\left[2\cos h\left(\frac{E_{f}}{2k_{B}T}\right)\right]+\frac{e^{2}}{4\hbar^{2}}\left[\frac{1}{2}+\frac{1}{\pi }\;\tan^{-1}\left(\frac{\hbar \omega -2E_{f}}{2k_{B}T}\right)-\frac{i}{2\pi }\ln\frac{{\left(\hbar \omega +2E_{f}\right)}^{2}}{{\left(\hbar \omega -2E_{f}\right)}^{2}+4{\left(k_{B}T\right)}^{2}}\right]$$

where *T*, *k_B_*, and *ħ* are the absolute temperature of the environment, Boltzmann constant, and reduced Planck’s constant, respectively, and *ω* is the angular frequency, *E_f_* is the Fermi energy level, and *τ* is the electron-phonon relaxation time. The first term in Equation (1) is derived from the intra-band transitions, and the second term is derived from the inter-band transitions. For the THz frequency domain (hω << 2*E_f_*) at room temperature, according to the Pauli exclusion principle, the inter-band transitions in the graphene are negligibly small; therefore, Equation (1) can be safely simplified to the Drude model [37]:

(2) $$\sigma_{gra}=\frac{e^{2}E_{f}}{\pi \hbar^{2}}\frac{i}{\left(\omega +i/\tau \right)}$$

It can be seen from Equation (2) that due to the existence of the carrier density, the carrier density can be changed by voltage or chemical doping, thereby adjusting the surface conductance *σ_gra_* of graphene through the Fermi level. The relationship between the Fermi level and the carrier density can be described by $E_{f}=\hbar V_{F}\sqrt{\pi n}$ [38]. The Fermi velocity of graphene *V_F_* is 1 × 10^6^ m/s, and *n* represents the carrier density of graphene. We set the values of the Fermi level and relaxation time of graphene as *E**_f_*** = 0.7 eV and *τ* = 1 ps, respectively. The ambient temperature at room temperature was fixed at 300 K. In this work, we used the finite-difference time-domain (FDTD) numerical simulation method to analyze the three-dimensional absorber structure in the frequency domain. Plane wave occurred perpendicularly along the *z*-axis. We used periodic boundary conditions in the x and y directions and perfectly matched layer (PML) boundary conditions in the z direction. During the simulation, the simulation time and grid accuracy were set to 40,000 fs and 5, respectively. The size of the simulation area was 15 µm × 15 µm, and the grid size in graphene was ∆*x* = ∆*y* = 0.15 µm, Δ*z* = 0.05 µm. In the calculation, a suitable non-uniform grid can be used to meet the conditions for good convergence results.

## 3. Results and Discussion

### 3.1. Single-Layer Graphene Metasurface Structure

Using the FDTD solutions, we studied a unit cell of a graphene-based THz absorber and obtained its electromagnetic response. The system model uses a plane wave as the light source. To investigate the absorption performance, the reflection and transmission spectrum of the absorber were captured. According to Kirchhoff’s current law, the relationship between the absorptivity *A*, the transmittance *T_tra_*, and the reflectance *R* is *A* = 1 – *T_tra_* − *R*. A high absorption can be achieved by minimizing *R* and *T_tra_* simultaneously. The transmission is almost equal to zero (*T_tra_* ≈ 0) in the total reflection geometry, so the absorption efficiency of the proposed absorber can be briefly expressed by *A* = 1 − *R*.

**Figure 2:**
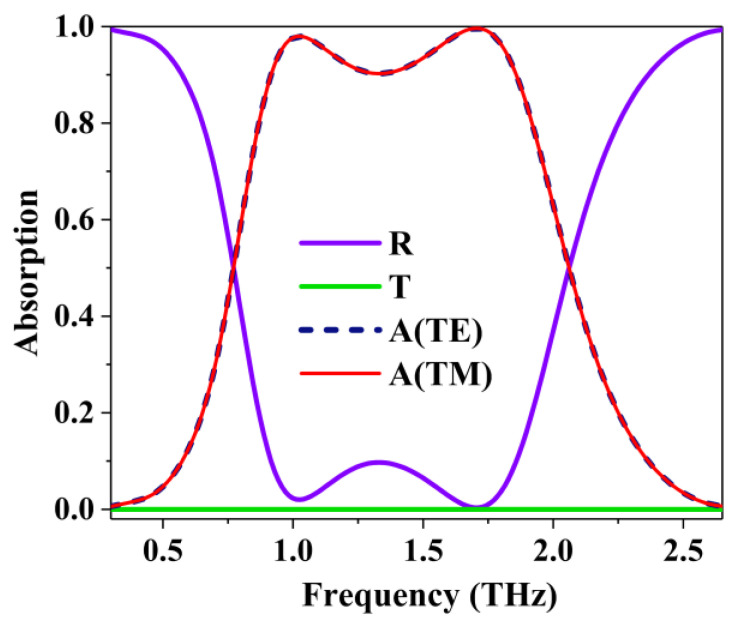
Transverse electric (TE) mode and transverse magnetic (TM) mode reflection (R), transmission (T), and absorption (A) spectra.

To study the absorption characteristics of the periodically annular porous patterned graphene structure proposed, we first simulated the spectral distribution under TE polarization and TM polarization conditions. Figure 2 shows the absorption spectra under TE and TM polarization conditions when the Fermi level of graphene is *Ef* = 0.7 eV with normal plane-wave incidence. As expected, due to the symmetry of the structure, the absorber exhibited the same absorption characteristics under TE and TM polarization states. The simulated results show that nearly perfect absorption with a maximum absorption of 99.6% at 1.70 THz can be achieved. There are clearly two absorption peaks, with an absorption rate of 98.0% and 99.6% at 1.02 THz and 1.70 THz, respectively. The 90% absorbance bandwidth from 0.91 THz to 1.86 THz is 0.95 THz. Fractional bandwidth, which is the absolute bandwidth relative to the center frequency, is approximately 68.6%.

**Figure 3:**
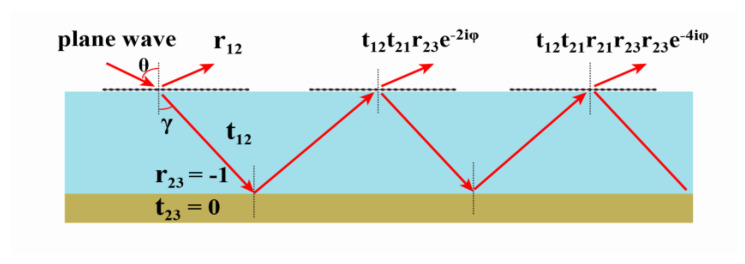
Schematic diagram of multiple reflections under normal incidence *x*-polarized.

The mechanism of the absorber is explained using Fabry–Perot interference theory. The structure of the absorber can be generally equivalent to a Fabry–Perot resonator, which is mainly composed of a partial reflector and a total reflector [39]. The graphene metasurface on the top of the absorber can be seen as a partial reflector, and the bottom metal plate of it can be regarded as a total reflector. Figure 3 shows the optical coupling in such a resonator. When a plane wave is incident perpendicularly along the *x*-axis polarization direction, the amplitude of the incident electromagnetic wave is represented by *E_inc_* and the amplitude of the reflected electromagnetic wave is represented by *E_ref_.* We can obtain the reflection coefficient of the metasurface absorber R as [40]:

(3) $$R=\frac{E_{ref}}{E_{inc}}=\frac{r_{12}+\left(t_{12}t_{21}-r_{12}r_{21}\right)r_{23}e^{i2\beta d}}{1-r_{21}r_{23}e^{i2\beta d}}$$

where *r*_12_ and *r*_21_ are the ratios of the reflected wave’s complex electric field amplitudes. Likewise, the transmission coefficients *t*_12_ and *t*_21_ are the ratios of the transmitted wave’s complex electric field amplitudes. The reflection coefficient of the bottom metal total reflection plate is *r*_23_ = −1, β = 2π*n*_2_/*λ*_0_ is the propagation constant, and *n*_2_ is the refractive index of the dielectric layer. According to the formula *A* = 1 − *R*, when *R* is equal to 0, the absorption rate *A* reaches the maximum value.

**Figure 4:**
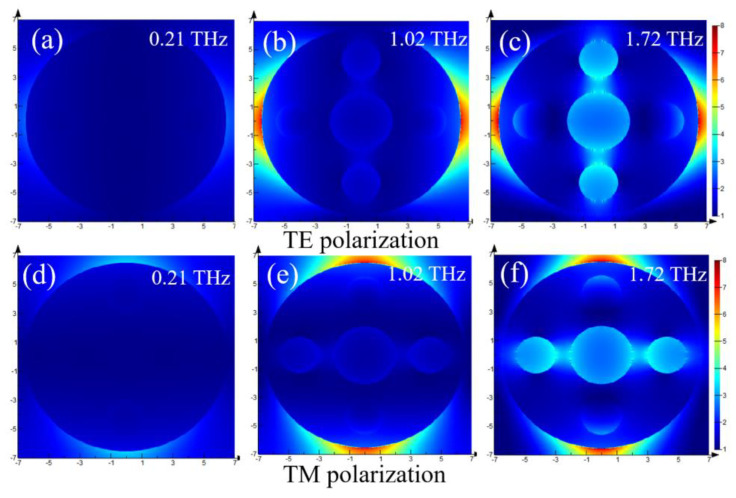
Simulated electric field amplitude (|E|) distributions of the proposed absorber, in TE polarization on *xy* plane at (**a**) 0.21 THz, (**b**) 1.02 THz, (**c**) 1.72 THz; in TM polarization on *xy* plane at (**d**) 0.21 THz, (**e**) 1.02 THz, (**f**) 1.72 THz.

Then, to further explore the absorption characteristics of the absorber, under the conditions of Fermi level *Ef* = 0.7 eV and plane-wave perpendicular incidence, we obtained the electric field amplitude (|E|) distribution of *f* = 0.21 THz, *f* = 1.02 THz, and *f* = 1.72 THz in TE and TM polarization modes, separately. Figure 4 shows the electric field amplitude distribution of the unit cell structure under two polarization conditions. Figure 4a–c show the TE electric field amplitude distributions of the unit cells on the xy plane at 0.21 THz, 1.02 THz, and 1.72 THz, respectively. Figure 4d–f show the TM electric field amplitude distributions of the unit cells on the xy plane at 0.21 THz, 1.02 THz, and 1.72 THz, respectively. For comparative analysis, we obtained the electric field amplitude distribution of the unit cell structure at 0.21 THz, 1.02 THz, and 1.72 THz. A strong electric field limit was found at 1.02 THz and 1.72 THz, and at 0.21 THz, corresponding to 2% absorbance; the electric field almost disappears. Due to the strong localized surface plasmon resonance of the patterned graphene structure, most of the electric field was limited to the edge of the ring-shaped graphene, and part of the electric field was distributed inside the ring for both polarizations. This phenomenon is caused by strong electric dipole resonance, which can effectively capture the energy of light, and it shows that strong electric field confinement will lead to higher absorption [41]. Because the proposed absorber has symmetry, the electric field amplitude distribution of TE polarization is the same as that of TM polarization electric field after 90° rotation, which corresponds to the same absorption spectrum of TE and TM polarization.

**Figure 5:**
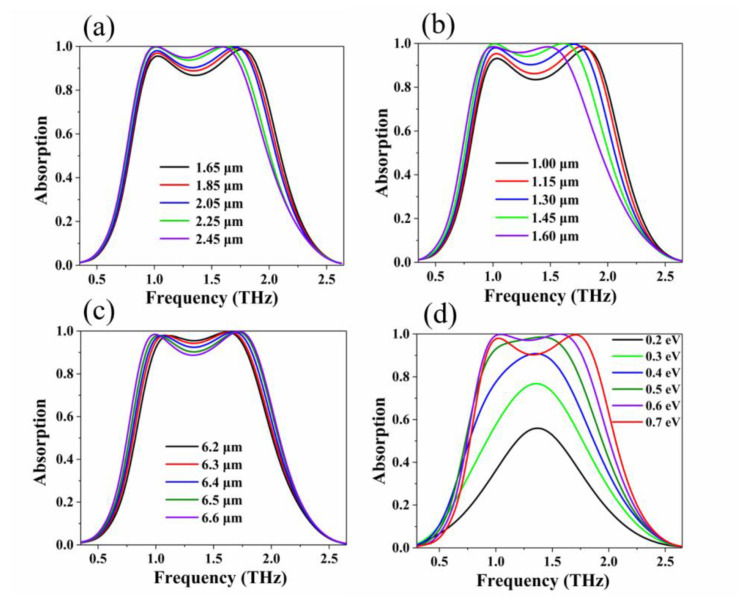
Simulated geometric parameters of terahertz wave absorber and graphene Fermi level. (**a**) The value of the inner circle radius *r* of the graphene ring, (**b**) the value of the small circle radius *p* within the graphene ring, (**c**) the value of the outer diameter *R* of the graphene ring, (**d**) Simulated absorption at different Fermi levels *E*_f_ from 0.2 eV to 0.7 eV of graphene.

Next, we investigated the influence of the geometric parameters of the absorber and the graphene Fermi level on the absorption spectrum. According to the influence of the geometric parameters of the structure on the absorption spectrum, the geometric parameters with the best absorption performance are obtained after optimization. When the geometric parameters of the structure have been adjusted, the variation of the absorption band has the same trend, due to the symmetry of the absorber. Figure 5a shows the influence of the geometric value of the inner circle radius *r* of the graphene ring on the absorption rate when other parameters of the absorber structure are kept at the optimal value under the normal incidence of the plane wave. When we only changed the value of the inner circle radius of the graphene ring (the inner circle radius *r* changed from 1.65 μm to 2.45 μm) and other parameters were unchanged (*p* = 1.3 μm, *R* = 6.5 μm, *E_f_* = 0.7 eV), the first absorption peak showed a slight decrease, and the absorption wave had traces of blue shift. When *r* was gradually reduced, the bandwidth of the absorption wave slowly increases, but when *r* was less than 2.05 μm, the absorption rate of the middle part of the absorption band was reduced to below 90%.

The influence of the value of the small circle radius *p* on the absorption spectrum is shown in Figure 5b. Because the symmetry of the structure is unchanged, the waveform of the absorption bandwidth has the same trend. The change of the absorption spectrum caused by the change of the small circle radius *p* is similar to the parameter *r*. As *R* gradually decreases, the first absorption peak slightly decreases. The absorption bandwidth gradually increases, and the second absorption peak shifts slightly toward high frequencies. Figure 5c shows the change of the absorption spectrum when the value of the radius *R* of the outer circle of the graphene ring is changed. When the change value of the outer radius *R* gradually decreases, the first absorption peak shifts slightly toward blue, and the second absorption peak tends to shift slowly toward red. Obviously, as the value of *R* decreased, the band with an absorption rate of 90% gradually decreases. When *R* = 6.6 μm, the absorption rate of part of the absorption broadband is less than 90%. Figure 5d shows a spectrum of the absorption rate with the change of the Fermi level of the graphene under normal incidence for the TE polarization. By increasing the Fermi level of graphene, the resonance intensity and local electric field of the plasmon resonance are enhanced, so the absorption of the absorber can be tuned. As graphene’s Fermi level *E*_f_ changes from 0.2 eV to 0.7 eV, the absorbance of the absorber can be adjusted from 55% to 100%. In particular, at Fermi level *E*_f_ = 0.6 eV, the absorption spectrum has two absorption peaks at almost 100%, at 1.04 THz and 1.5 THz. At this time, the absorption characteristics of graphene begin to become saturated. At Fermi level *E*_f_ = 0.7 eV, the absorption rate of the absorber decreases, but there is a wider fractional absorption bandwidth. Graphene-based terahertz photoelectric devices such as absorbers, sensors, and detectors have the characteristics of small size and excellent performance. These are conducive to device integration and application.

**Figure 6:**
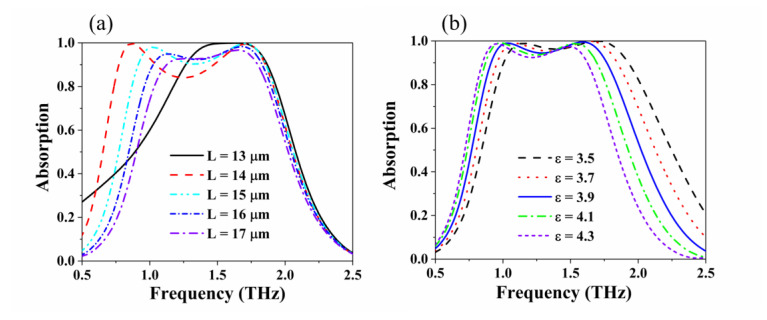
Results of simulating the periodic unit of the structure and relative permittivity of the dielectric layer. (**a**) Influence of the size of the periodic unit (*L*) of the structure on the absorption performance. (**b**) Effect of the relative dielectric constant of the dielectric layer (*ε*) on absorption performance.

Then, we studied the effect of the size of the periodic unit (*L*) and the relative permittivity (*ε*) of the dielectric layer on the absorption performance of the absorber. The simulated results are shown in Figure 6. Figure 6a shows that when the period *L* increases, the absorption bandwidth gradually decreases, and the left absorption peak begins to decrease. The absorption rate of the absorber reaches 99% in the range of 1.44 Thz to 1.73 THz when *L* = 13 μm, which is equal to the radius of the outer ring of the graphene ring. Figure 6b shows the effect of relative permittivity (*ε*) of the dielectric layer on the absorber. As the relative permittivity gradually increases, the absorption bandwidth changes in small shifts to lower frequencies. The relative permittivity of the dielectric layer has little effect on the waveform of the absorption bandwidth of the absorber.

**Figure 7:**
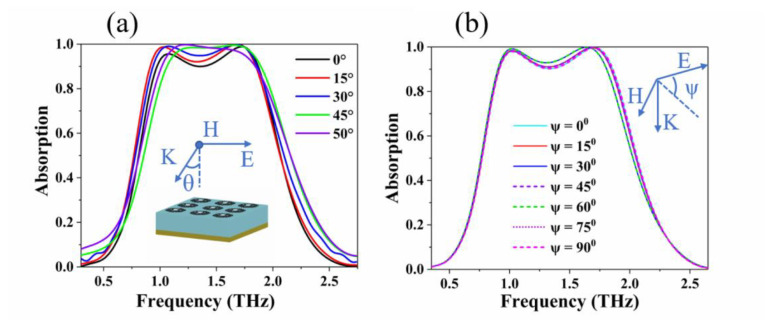
(**a**) Relationship between the absorption spectrum and the angle of incidence *θ* under TE polarization. (**b**) Dependence of the absorption spectrum on the polarization angle *ψ* at normal incidence.

Finally, we studied the dependence of the absorber on the incident angle of the light source and the behavior of the polarized incident angle. The graphene ring was designed in an axis-symmetrical pattern, so that the absorber structure could eliminate polarization sensitivity. Figure 7a shows the dependence of the absorption spectrum on the polarization angle *θ* when the light source is obliquely incident under TE polarization conditions. It can be seen from the figure that the designed absorber has good absorption performance and relatively stable broadband absorption rate in a wide range of incident angles. It can be seen that the broadband absorption under TE polarization conditions at a light source incident angle *θ* = 50° still reaches more than 90% in the range of 1.01 THz to 1.84 THz. The absorber has advantages, such as a wide normalized bandwidth or polarization insensitivity and wide-angle incidence. Figure 7b shows the absorption spectrum when the polarization angle *ψ* changes from 0° to 90° at normal incidence. From the figure, when the polarization angle changes from 0° to 90° under normal incidence, the absorption broadband of the absorber does not change much with the polarization angle *ψ*. With the increase of the polarization angle *ψ*, the absorption broadband characteristics can be kept substantially unchanged, due to the axisymmetric characteristics of the absorber. In future practical applications, the terahertz broadband absorber must have wide-angle incidence and polarization insensitivity.

### 3.2. Double-Layer Graphene Metasurface Structure

**Figure 8:**
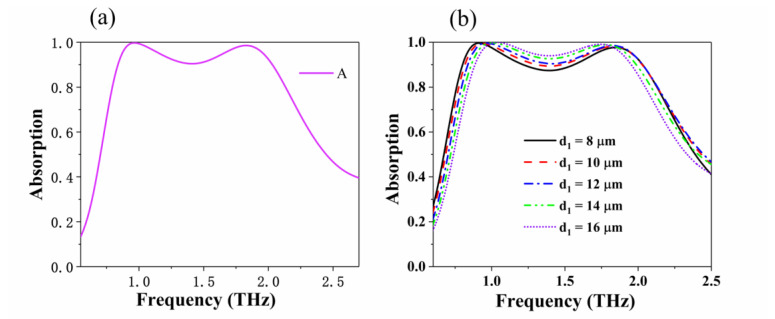
(**a**) Absorption spectrum of a proposed double-layer graphene absorber. (**b**) Absorption spectrum of the proposed double-layer graphene interlayer thickness *d*_1_.

An absorber with better absorption performance can be obtained by applying a multilayer stack structure. Here, the double-layer graphene metasurface is isolated by silica, and a model diagram of it is shown in Figure 1c. Double-layer graphene metasurface is divided by SiO_2_ with thickness *d*_1_ = 12 μm, the value of *d*_2_ is set to 16 μm, and the value of the graphene Fermi level *E*_f_ is set to 0.6 eV. Other geometric parameters (*R* = 6.5 μm, *r* = 2.05 μm, *p* = 1.3 μm) are consistent with the single-layer graphene absorber. Figure 8a shows the absorption spectrum of a double-layer graphene structure at normal incidence. The results show that 90% of the absorption bandwidth reaches 1.21 THz from 0.83 THz to 2.04 THz. The fractional bandwidth (the absolute bandwidth relative to the center frequency) is approximately 84.3%. Compared with the single-layer graphene absorber, the absorption bandwidth is extended by 0.21 THz, and the fractional bandwidth is increased by 15.7%. We studied the relationship between the interval thickness d1 and the absorption spectrum, and only changed it from 8 μm to 16 μm. Figure 8b shows the relationship between different interlayer thicknesses and absorption spectra: The absorption rate of the middle of the absorption band is less than 90% when d1 does not exceed 12 μm. The bandwidth will decrease when *d*_1_ increases, whereas the absorption rate of the middle of the absorption band will increase. Thus, there is an optimal d1 for the absorption rate and bandwidth. This is because the strength of the resonance coupling on the surface of the upper and lower layers of graphene depends, to a large extent, on the distance between them. Increasing or decreasing the coupling distance will reduce the near-field coupling to some extent, thus showing changes in absorption performance.

## 4. Conclusions

In this paper, we have proposed an active tunable, wide-angle, and polarization-insensitive broadband terahertz absorber, based on a ring-porous patterned graphene metasurface. The simulated results show that the best characteristics can be achieved by optimizing the geometric parameters of the absorber. They also show that the bandwidth of the absorber is over 0.95 THz (with a fractional bandwidth of 68.6%), for 90% absorbance under normal incidence. It is verified by simulation that the symmetrical structure of the absorber has the characteristics of wide-angle incidence and polarization independence. By changing the Fermi level of graphene, the absorption rate of the absorber can be flexibly adjusted. The absorber after coupling two layers of graphene structure obtains a fractional bandwidth of 84.3%. We think the absorber has potential applications in the fields of sensing, detection, and other optoelectronic devices, such as those used as an absorber in a Q-switched laser.
